# Supplementary material for: Hybrid Models and Biological Model Reduction with PyDSTool
Source: PLoS Comput Biol. 2012 Aug 9;8(8):e1002628. doi: 10.1371/journal.pcbi.1002628 (PMC3415397; doi:10.1371/journal.pcbi.1002628)
Supplement: Text S4 — Complete source code for the PyDSTool package (version 0.88.120504). Includes API documentation and help files linking to web pages. This file is identical to the current public release on Sourceforge.net. (ZIP) [file pcbi.1002628.s004.zip › PyDSTool/html/PyDSTool.integrator'-module.html]

xml version="1.0" encoding="ascii"?


PyDSTool.integrator'


| Home | Trees | Indices | Help | PyDSTool |
| --- | --- | --- | --- | --- |

Package PyDSTool ::
Module integrator'

[hide private]

[frames] | no frames]

# Module integrator'

source code

Basic integrator interface class Erik Sherwood, September 2006


|  |  |
| --- | --- |
| Classes | [hide private] |

integrator


|  |  |
| --- | --- |
| Variables | [hide private] |

Inf = `inf`

\_all\_int = `(<type 'int'>, <type 'numpy.integer'>, <type 'numpy...`

\_real\_types = `(<type 'int'>, <type 'numpy.integer'>, <type 'fl...`

isinf = `<ufunc 'isinf'>`


|  |  |
| --- | --- |
| Variables Details | [hide private] |

### \_all\_int

Value:
:   ```
    (<type 'int'>,
     <type 'numpy.integer'>,
     <type 'numpy.int32'>,
     <type 'numpy.int32'>,
     <type 'numpy.int8'>,
     <type 'numpy.int16'>,
     <type 'numpy.int32'>,
     <type 'numpy.int64'>)
    ```

### \_real\_types

Value:
:   ```
    (<type 'int'>,
     <type 'numpy.integer'>,
     <type 'float'>,
     <type 'numpy.floating'>)
    ```

  


| Home | Trees | Indices | Help | PyDSTool |
| --- | --- | --- | --- | --- |

|  |  |
| --- | --- |
| Generated by Epydoc 3.0.1 on Fri May 4 15:24:05 2012 | http://epydoc.sourceforge.net |
